# The effects of video-based exergames on balance and agility in children with and without developmental coordination disorder: a meta-analysis

**DOI:** 10.7717/peerj.21664

**Published:** 2026-09-02

**Authors:** Lei Zhao, Jianing Wang, Haiwei Shang, Ping Liu, Jilan Zhou, Yushuang Cheng

**Affiliations:** 1 Department of Physical Education, Qingdao Huanghai University, Qingdao, China; 2 Health Check-up Center, Huangdao District Central Hospital of Qingdao, Qingdao, China; 3 School of Health Sciences, Universiti Sains Malaysia, Kubang Kerian, Malaysia; 4 School of Physical Education, Jinan Preschool Education College, Jinan, China

**Keywords:** Video-based exergames, Developmental coordination disorder, Children, Balance, Meta-analysis

## Abstract

**Background:**

Developmental coordination disorder (DCD) is a relatively common neurodevelopmental disorder that affects 5–6% of school-age children worldwide. This study evaluated the effects of video-based exergames on balance and agility in children with and without DCD.

**Methodology:**

Four databases were searched, and the inclusion criteria include: 1. Children with DCD under 14 years old. 2. Randomized controlled trials. 3. The experimental group (EG) used video-based exergames. 4. Control group (CG) did not use exergames or typically developing (TD) children. 5. The test indicators before and after intervention included balance and agility. Use the Cochrane bias risk assessment tool to evaluate the quality of the selected study. Select standardized mean difference (SMD) as the appropriate effect scale index, and use Revman 5.4 software to analyze the mean difference of the selected article data (Registration number: CRD420261289081).

**Results:**

The meta-analysis outcomes showed video-based exergames considerably enhanced the balance (SMD, 0.82 (0.53, 1.11), *p* < 0.05, *I* ^2^ = 45%) of children with DCD, rather than agility [SMD, 0.93 (–0.28, 2.14), *p* = 0.13, *I* ^2^ = 86%] compared to the no exergames group. The balance (SMD, 0.84 (0.61, 1.07), *p* < 0.05, *I* ^2^ = 0%) and agility [SMD, 0.47 (0.15, 0.78), *p* < 0.05, *I* ^2^ = 0%] of children with DCD of TD children were also considerably enhanced with exergames interventions.

**Conclusions:**

Video-based exergames present potential benefits for improving the balance of children with DCD. Consequently, such interventions could be a potential non-pharmacological treatment for children with DCD.

## Introduction

Affecting between 5% and 6% of school-aged children worldwide, developmental coordination disorder (DCD) is a relatively common neurodevelopmental condition (Romeo et al., 2022). The illness is characterised by persistent motor coordination and skill acquisition issues, with core deficits including balance control and agility impairments, which are foundational and vital for daily activities (such as walking and climbing), participation in physical education and sports, and overall physical health (Promsri, 2022). Consequently, children suffering from DCD frequently exhibit reduced static or dynamic balance, slower direction-changing abilities, and increased fall risk than their typically developing (TD) peers (Scott-Roberts & Purcell, 2018). These symptoms contribute to sedentary behaviour, low physical fitness, and negative psychosocial outcomes (Lau et al., 2015).

Traditional motor interventions aiming at improving the balance and agility of children with DCD have demonstrated a certain level of efficacy. Nevertheless, the effectiveness of these strategies is frequently limited by low adherence, particularly among children with DCD, due to their repetitiveness and insufficient motivational elements (Zwicker, Harris & Klassen, 2013; Smits-Engelsman et al., 2018). Video-based exergames are active video games that integrate whole-body movement with virtual reality (VR) or motion-sensing technology. These games are emerging as a promising alternative to conventional motor treatment strategies.

Several exergames, such as those developed by Nintendo Wii Fit and Xbox Kinect, offer unique advantages, including immediate visual or auditory feedback, adaptability to individual performance, and enhanced engagement through gamification. Therefore, these video games are especially appealing to children (Ferguson et al., 2013; Jelsma et al., 2014). Consequently, exergames provide a novel intervention model to enhance the physical function of DCD and TD children.

In a meta-analysis report, the outcomes of Wii-based treatment approaches on the balance and agility of children with DCD were assessed. Nonetheless, the review only included six studies and only compared the influence of Wii on children with DCD to conventional care. The report also excluded other exergame intervention modes and TD children. Available literature also does not provide a clear association between video-based exergames and the balance and sensitivity of children with and without DCD. Nevertheless, several studies (Jelsma et al., 2016, 2023) indicated that exergames can improve the balance and agility of children with DCD more effectively compared to their counterparts. Meanwhile, other studies revealed no notable variations among the participants in both groups. Accordingly, this study adopted a meta-analysis method to evaluate the influence of video-based exergames on balance and agility in children with and without DCD.

## Materials and Methods

### Search strategy

**Figure 1:**
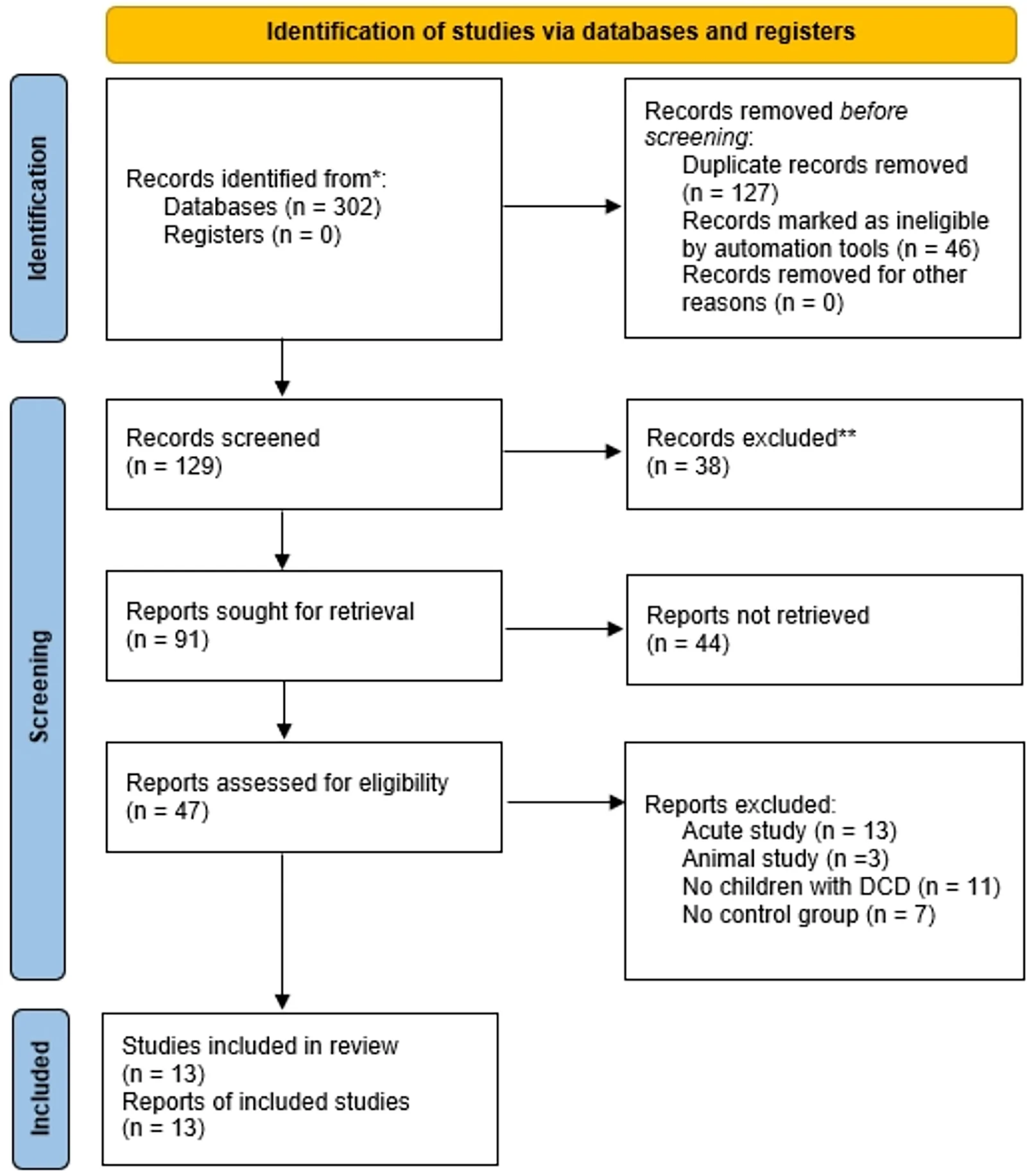
Flow diagram of the search results using the preferred reporting items for systematic reviews and meta-analysis (PRISMA).

This systematic review was performed in accordance with the PRISMA (Page et al., 2021) guidelines (Fig. 1). This review was also registered on PROSPERO (CRD420261289081). The Web of Science, PubMed, SCOPUS, and EBSCO databases were searched for randomised controlled trials (RCTs), clinical trials, and pilot studies with several keywords combinations, such as “exergame”, “children”, and “developmental coordination disorder”.

The databases were searched by two independent researchers (L.Z and J.W) while a third researcher (Y.C) was tasked with mediating any discrepancies. Only articles published between 1^st^ January, 2006 and 30^th^ January, 2026 were considered during the process. Subsequently, a reference list of the identified publications was searched manually to determine additional eligible reports. File S1 details the information regarding the search strategy applied.

### Inclusion and exclusion criteria

In this review, full-text English articles, targeting RCTs on the influence of video-based exergames intervention on balance, agility, aiming and catching (AC) and manual dexterity (MD) of children with DCD were reviewed. Reports that recorded measurements before and after interventions for comparison, utilised video-based exergames for the experimental group (EG) and no exergames or TD children as the control group (CG), and reported assessed parameters as mean (standard deviation) or median (interquartile range) were included. Meanwhile, abstracts, conference proceedings, grey literature, and poster presentations were not considered for review.

### Quality assessment

In this meta-analysis review, two validated tools were integrated for quality assessment to ensure methodological rigour. First, the overall quality of each outcome estimate was rated based on the GRADE method. Four hierarchical levels, high, moderate, low, and very low, were applied during this phase. Each level was determined by five critical dimensions: inconsistency, imprecision, risk of bias, indirectness, and additional considerations. Subsequently, the Cochrane bias risk tool (Higgins et al., 2019) was adopted to systematically determine components, including the bias risk of selected articles, covering random sequence synthesis, allocation concealment, participants, personnel, and outcome evaluation blinding, incomplete outcome data, and selective reporting (Li et al., 2023). Each assessment item was then categorised with “Yes”, “No”, or “Unclear” indicators.

### Data extraction

The data in this study were extracted by researchers L.Z and J.W on 2^nd^ February, 2026, with any disagreements resolved with the assistance of another researcher, Y.C Following the retrieval of data from each selected article, they were systematically organised into a tabular format (Table 1) according to various primary variables, including participants’ age, gender, duration, exergames protocol, and control group protocol. The corresponding authors were contacted to request supplementary information for articles with missing data. If data were still not provided, the article was exempted from further analysis.

**Table 1:** Characteristics of included studies.

Study	Age (y)	Gender	Duration	Exergaming protocol	Control group	Index
Harkness-Armstrong et al. (2025)	EG: 9.0 ± 1.0 CG: 9.0 ± 1.0	12 M/5 F	6 weeks 3×/week	Switch; challenge balance, and focus on strengthening the lower limbs and core; 30 min.	No intervention	BA
Ashkenazi et al. (2013)	5.2 ± 0.6	25 M/5 F	12 weeks 10×/week	Play station 2; VR exercise, goal based upper limb movements; 60 min.	Physical therapy	AC, BA, MD
Smits-Engelsman, Jelsma & Ferguson (2017)	EG: 8.2 ± 1.1 CG: 8.0 ± 1.2	18 M/17 F	5 weeks 2×/week	Wii Fit; functional strength, anaerobic fitness, balance skills and agility; 20 min.	TD children	AG, BA
Draghi et al. (2024)	EG: 9.0 ± 1.1 CG: 9.6 ± 1.0	37 M/39 F	5 weeks 2×/week	Xbox; river rush, rally ball, 20,000 leaks, reflex ridge, and space pop; 20 min. Wii Fit; functional strength, anaerobic fitness, balance skills and agility; 20 min.	TD children	BA
Smits-Engelsman et al. (2015)	EG: 7.9 ± 1.2 CG: 7.7 ± 1.1	18 M/16 F	5 weeks 2×/week	Wii Fit; ski slalom game for dynamic balance control; 60 min.	TD children	BA
Smits-Engelsman, Bonney & Ferguson (2021)	EG: 9.3 ± 1.2 CG: 9.8 ± 1.4	28 M/32 F	10 weeks 2×/week	Wii Fit; functional strength, anaerobic fitness, balance skills and agility; 30 min.	TD children	AC, AG, BA, MD
Hammond et al. (2014)	EG: 8.5 ± 1.1 CG: 9.5 ± 1.4	14 M/4 F	4 weeks 3×/week	Wii Fit; games that focus on balance and coordination; 10 min.	Treatment as usual	AC, AG, BA, MD
Jelsma et al. (2014)	EG: 8.2 ± 1.4 CG: 8.5 ± 1.1	29 M/19 F	6 weeks 3×/week	Wii Fit; ski slalom game for dynamic balance control; 30 min.	No intervention	AG, BA
Jelsma et al. (2016)	9.3 ± 3.7	22 M/12 F	6 weeks 3×/week	Wii Fit; ski slalom game for dynamic balance control; 30 min.	No intervention; TD children	BA
Jelsma et al. (2023)	EG: 8.9 ± 1.2 CG: 9.8 ± 1.2	34 M/34 F	5 weeks 2×/week	Xbox; river rush, rally ball, 20,000 leaks, reflex ridge, and space pop; 20 min. Wii Fit; ski slalom game for dynamic balance control; 20 min.	TD children	AG, BA
Ju et al. (2018)	EG: 6.8 ± 1.3 CG: 7.0 ± 1.5	18 M/18 F	4 weeks 3×/week	iBalance; video games for training static and dynamic balance; 45 min.	No intervention; TD children	BA
Mombarg, Jelsma & Hartman (2013)	EG: 9.5 ± 1.8 CG: 9.7 ± 1.1	23 M/6 F	6 weeks 3×/week	Wii Fit; ski-jump, segway circuit, obstacle course and skate boarding; 30 min.	No intervention	AG, BA
Straker et al. (2015)	11.0 ± 1.0	10 M/11 F	16 weeks 4×/week	Play station 3; video game of upper and lower limb movements; 20 min.	Sedentary games	AC, BA, MD

**Note:**

EG, Exergaming group; CG, Control group; M, Male; F, Female; TD, Typically developing; AC, Aiming and Catching; AG, Agility; BA, Balance; MD, Manual Dexterity.

### Sensitivity analysis

Two methods were employed during sensitivity assessment: exclusion by individual study and switching to different statistical models.

### Statistical analyses

This study employed Review Manager (version 5.4.1; The Cochrane Collaboration, Copenhagen, Denmark) for analysing all information obtained. This review also adopted the methodology outlined in the Cochrane Handbook for Systematic Reviews of Interventions (Zhang et al., 2024) for assessing data based on the information available in the articles in cases where an effect size of interest for each outcome was not presented.

Although continuous variables represented the findings in the articles reviewed, each report adopted a different evaluation technique. Therefore, the standardised mean difference (SMD) denoted the effect scale index in this study. The methodology reported by Hozo, Djulbegovic & Hozo (2005) was also applied to convert median (range) to mean (standard deviation), when the outcomes contained medians (range) (Principles of Data Conversion: http://vassarstats.net/median_range.html).

This review determined the heterogeneity of the reviewed articles with the *I*^2^ statistic. A lower heterogeneity was denoted by a reduced *I*^2^, while non-heterogeneous reports were represented by *I*^2^ values under 50%, necessitating a fixed effect model. Meanwhile, *I*^2^ values equal to or over 50% indicated heterogeneity among the reviewed articles, which implied that a random effect model was required (Li et al., 2021). Finally, this study employed funnel and forest plots with uncertainty quantified at 95% confidence intervals (CI) to establish publication bias and SMD, respectively.

## Results

### Eligibility of studies

This review imported 302 articles identified from Web of Science, Scopus, PubMed, and EBSCO databases into Mendeley Desktop software (version 1.19.8). Following deduplication, 129 publications remained, which were further reduced to 91 articles after the titles and abstracts were subjected to a preliminary screening. Another 47 reports were removed following review of their full-text. Consequently, 13 articles were included in the final analysis. Figure 1 illustrates the comprehensive literature selection flowchart.

The respective institutions provided ethical approval for all protocols employed in the articles selected in this study. Two investigators (L.Z and J.W) also calibrated the consistency of the assessments of the reports. The procedure recorded a 0.946 Cohen’s kappa coefficient (*k*).

The final eight articles reviewed in this study involved 288 male and 218 female participants. Six reports also had a no-exergame group as the control group, while five studies had a group of TD children. In another two studies, no exergame and TD child groups were included as control groups. The reports were also conducted between four and 16 weeks.

### Quality assessment

**Figure 2:**
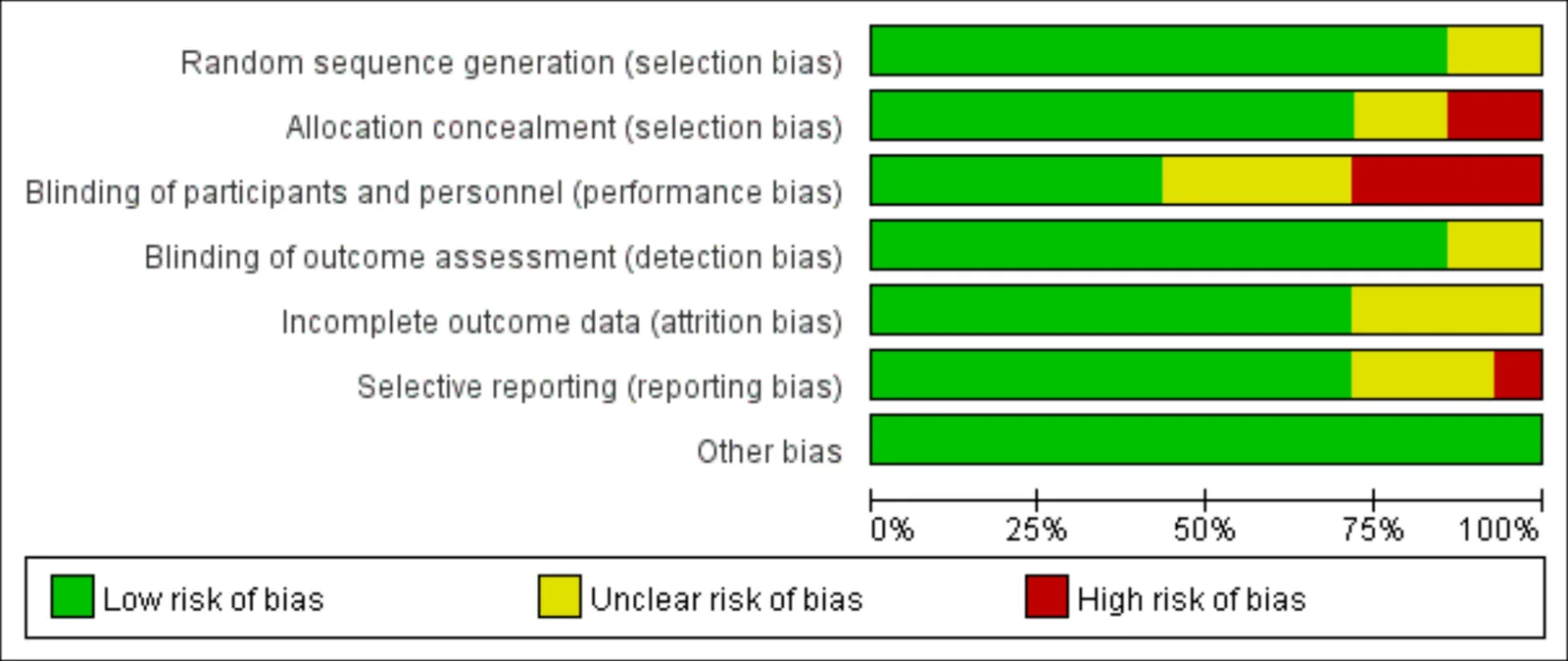
Analysis of risk of bias according to the Cochrane collaboration guideline.

Based on the methodological quality and potential bias risk results of the selected articles illustrated in Fig. 2, the overall quality was comparatively substantial, with proportions of low, high, and unclear bias risks at 75.51%, 7.14%, and 17.35%, respectively.

### Sensitivity analysis

During the sensitivity evaluation performed in this study, the model category was modified, and the maximum effect size was excluded. Resultantly, most outcomes were not notably altered following the modifications of various conditions, confirming the reliability of the research results. However, data obtained from comparing exergames with no exergames in sensitive indicators were unstable, and their statistical significance changed after switching models.

### Quantitative synthesis

**Figure 3:**
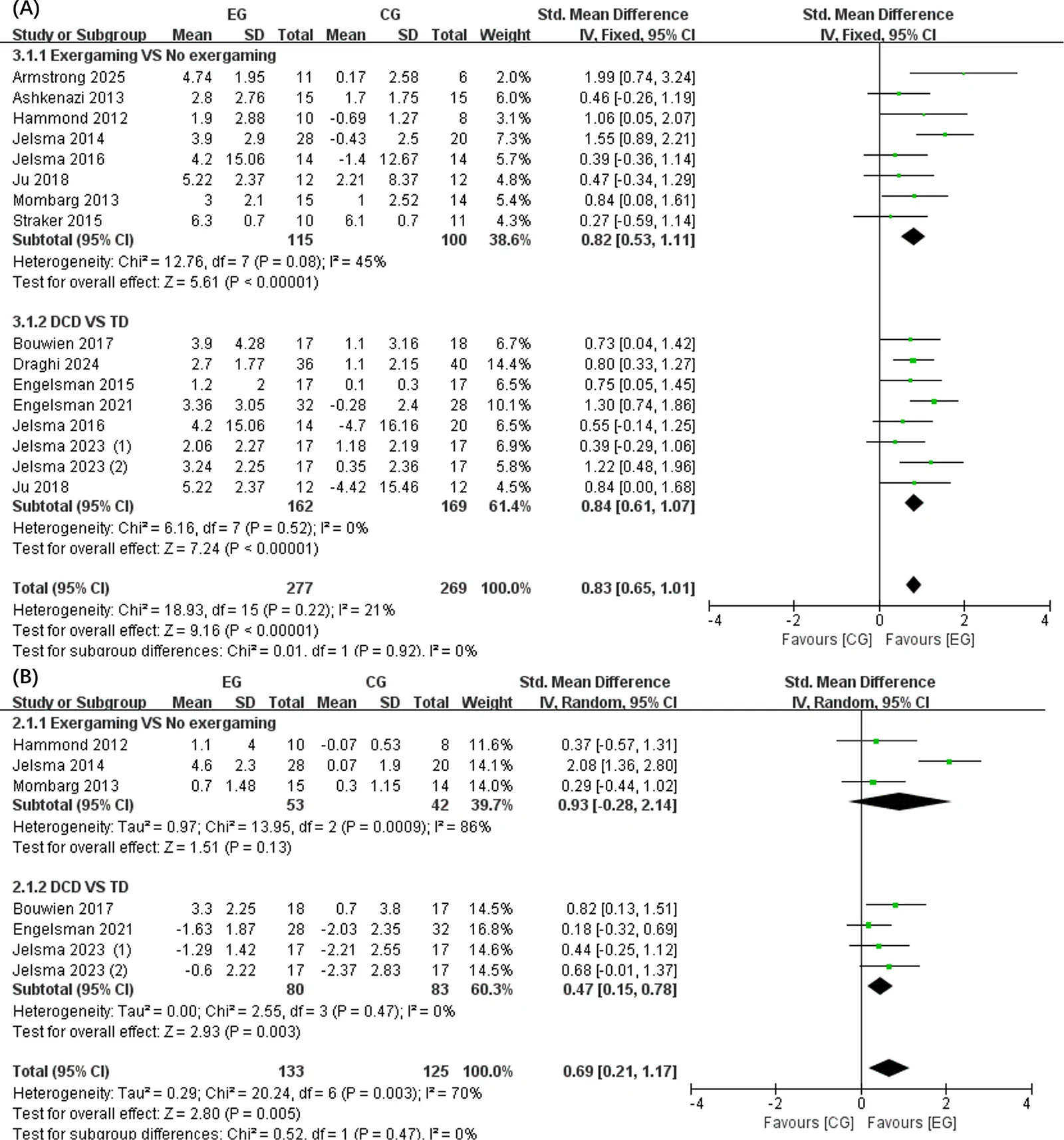
Forest plot illustrates the effects of EG *vs*. CG intervention on balance (A) and agility (B).

Eight (Ashkenazi et al., 2013; Mombarg, Jelsma & Hartman, 2013; Hammond et al., 2014; Jelsma et al., 2014, 2016; Straker et al., 2015; Ju et al., 2018; Harkness-Armstrong et al., 2025) and three (Ashkenazi et al., 2013; Hammond et al., 2014; Straker et al., 2015) articles reviewed the effects of exergames and no exergames on balance and agility in children with DCD, respectively, while the influence of exercise game strategies on balance and agility in children with DCD and TD was compared in seven (Smits-Engelsman et al., 2015; Jelsma et al., 2016, 2023; Smits-Engelsman, Jelsma & Ferguson, 2017; Ju et al., 2018; Smits-Engelsman, Bonney & Ferguson, 2021; Draghi et al., 2024) and three (Ashkenazi et al., 2013; Hammond et al., 2014; Straker et al., 2015) reports. Based on the results, exergames intervention enhanced the balance [SMD, 0.82 (0.53, 1.11), *p* < 0.05, *I*^2^ = 45%] of children with DCD, rather than agility [SMD, 0.93 (–0.28, 2.14), *p* = 0.13, *I*^2^ = 86%] compared to the no exergames group. The balance [SMD, 0.84 (0.61, 1.07), *p* < 0.05, *I*^2^ = 0%] and agility [SMD, 0.47 (0.15, 0.78), *p* < 0.05, *I*^2^ = 0%] of children with DCD and TD were also considerably enhanced with exergames interventions (Fig. 3).

**Figure 4:**
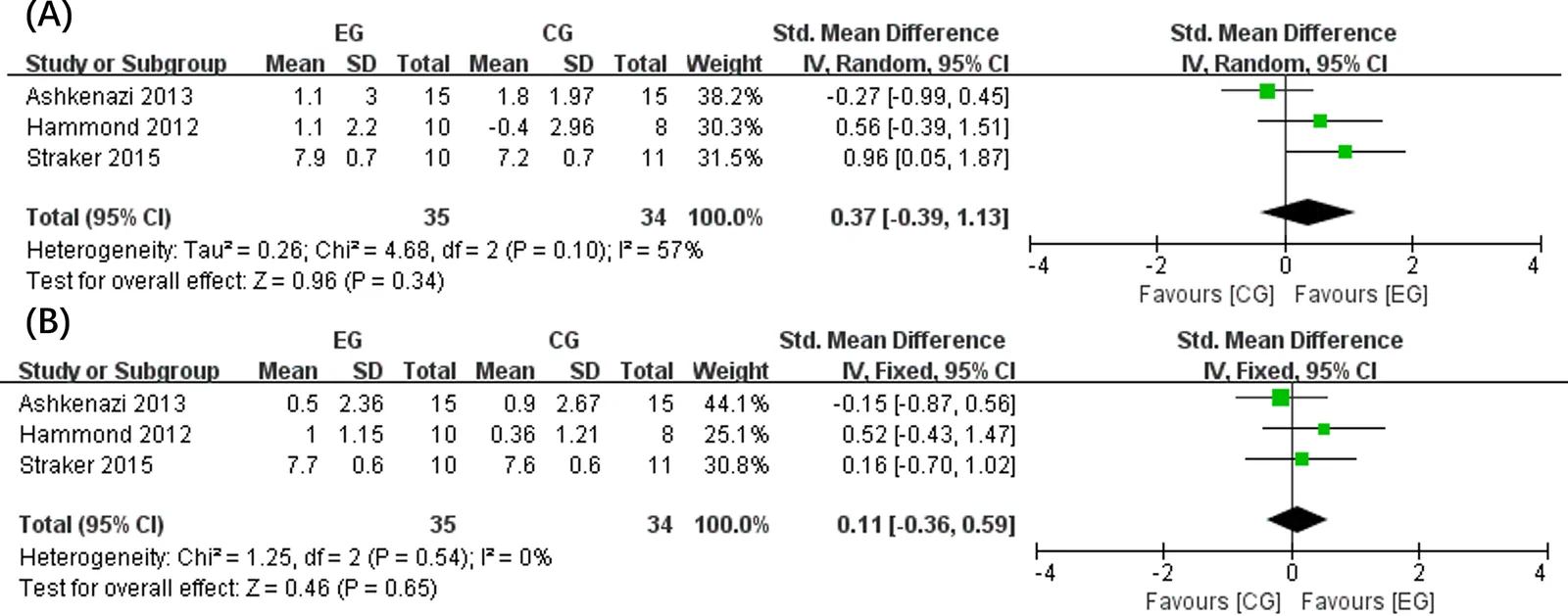
Forest plot illustrates the effects of video-based exergames on aiming and catching (A) and manual dexterity (B) before and after intervention.

Regarding secondary indicator assessment, three studies (Ashkenazi et al., 2013; Hammond et al., 2014; Straker et al., 2015) established the effects of exergames and no exergames on AC and MD in children with DCD, respectively. The outcomes also revealed that compared to the no exergames group, exergames interventions had no substantial influence on AC [SMD, 0.37 (−0.39, 1.13), *p* = 0.34, *I*^2^ = 57%] and MD [SMD, 0.11 (−0.36, 0.59), *p* = 0.65, *I*^2^ = 0%] of children with DCD (Fig. 4).

### Analysis of publication bias

**Figure 5:**
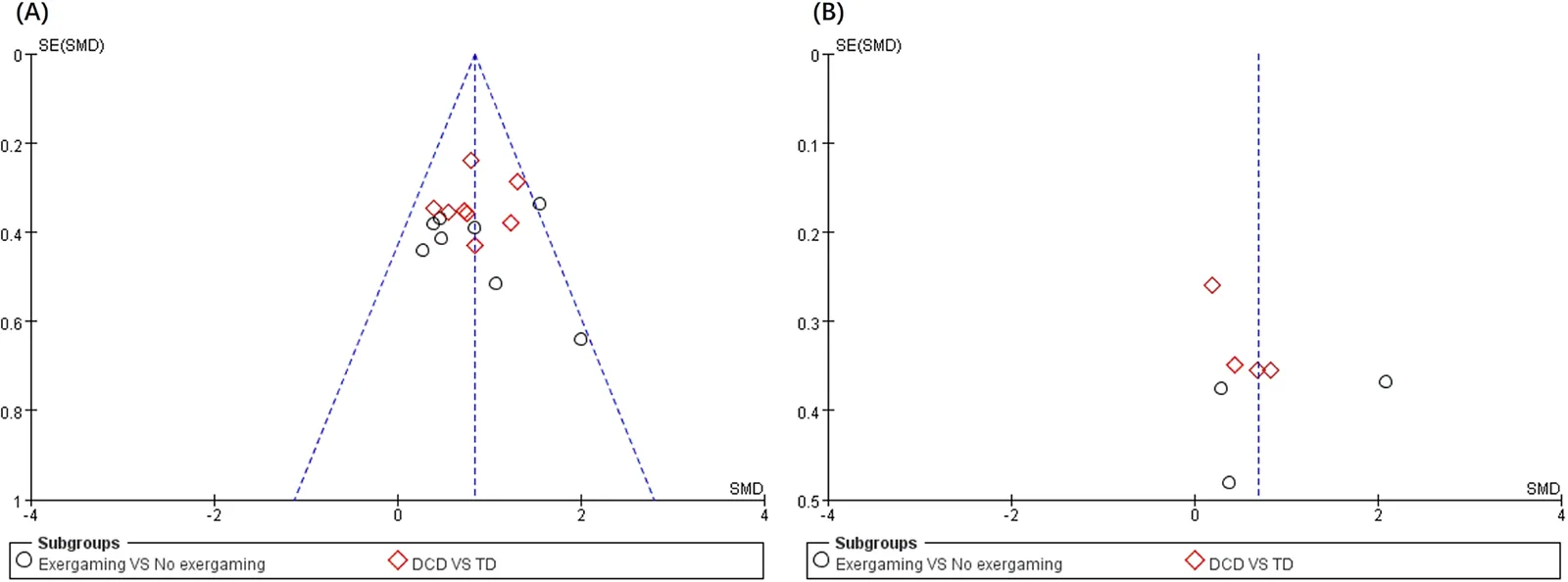
Funnel plot of publication bias for balance (A) and agility (B) in the EG *vs*. CG intervention.

**Figure 6:**
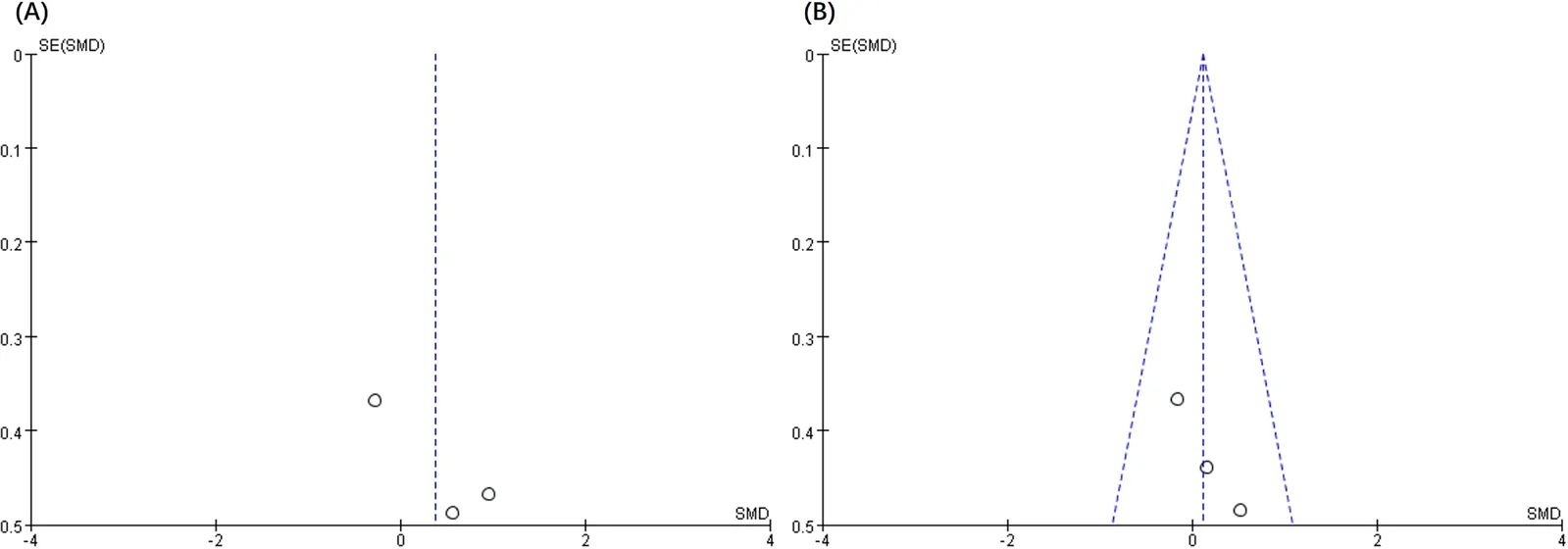
Funnel plot of publication bias for aiming and catching (A) and manual dexterity (B) in the video-based exergames.

This review employed a funnel plot to assess publication bias of the reviewed articles. The publications were assessed to a certain extent, considering that the total number of selected articles approached the minimum threshold for utilising a funnel plot (Lu et al., 2020). Based on the left-right symmetrical distribution demonstrated in Figs. 5 and 6, a slight likelihood of publication bias was observed.

## Discussion

This review established the outcomes of video-based exergame treatment approaches on the balance and agility of children with and without DCD. The differences in the effects of exergames and non-exergames on DCD and TD children were also highlighted. According to the outcomes, exergame interventions considerably enhanced balance compared to non-exergame strategies. Nevertheless, the agility between AC and MD did not vary notably following being subjected to the interventions. Exergames can also substantially improve the balance and agility in MD children compared to their TD counterparts. These observations indicated that exergames offer an interesting and promising intervention alternative for children with DCD.

The data procured in this meta-analysis confirmed that exergames consistently and positively improved the balance function in children with DCD, supporting the outcomes of multiple targeted experimental studies. In a study (Ashkenazi et al., 2013), a significant improvement in the balance subscale scores of the movement assessment battery for children (MABC) of children with DCD (ES = 1.26) was recorded after 10 weeks of Sony PlayStation 2 EyeToy intervention. The children in the study demonstrated a notable rise in single-leg standing time and centre of gravity movement speed. Interventions with Wii Fit devices also demonstrated similar results.

In an RCT (Jelsma et al., 2014) involving 48 children with DCD, the Bruininks-Oseretsky test of motor proficiency (BOT-2) balance standard score and dynamic balance control ability (such as centre of gravity adjustment speed in skiing slalom tasks) of the participants were measured. After 6 weeks of Wii Fit dynamic balance training, the children demonstrated significantly improved outcomes. At its core, Wii Fit continuously disturbs the centre of gravity (COP), stimulates the integration of proprioceptive and visual movements, and promotes the optimisation of posture control strategies, contributing to the positive observations.

The mechanism by which Exergames enhance balance in children with DCD remains unclear. Nonetheless, multisensory integration and real-time feedback regulation could explain the benefits of these types of video games. Other devices, such as Wii Fit and Nintendo Switch, also provide visual feedback, including screen posture prompts and proprioceptive stimuli, such as balance plate pressure sensing through dynamic tasks. Therefore, the efficiency of visual proprioceptive vestibular integration is optimised (Harkness-Armstrong et al., 2025), facilitating the rapid perception of posture deviations and correct strategies. The advantageous attributes of such devices can also shorten the activation latency of balance-related muscles, including the gastrocnemius, resulting in neuromuscular control patterns similar to those of typical developing children.

Neuromuscular control remodelling and regular training utilising exergames can stimulate the reorganisation of motor cortex function and improve balance-related neural pathway connections (Jelsma et al., 2023), muscle co-activation efficiency, and posture adjustment flexibility when the centre of gravity is disturbed. Such interventions also lay a physiological foundation for improving balance. Moreover, training compliance is considerably enhanced through effective training accumulation driven by motivation, fun, real-time rewards, and progressive difficulty design of games (Smits-Engelsman, Jelsma & Ferguson, 2017). Consequently, effective training dosage is ensured and the automation of balanced skills formation is promoted. Issues arising from insufficient exercise volume due to insufficient motivation in traditional training can also be avoided, leading to elevated stability.

Based on the outcomes documented in this study, exergames could significantly improve the sensitivity of children with DCD and narrow the gap with TD children, aligning with the data reported by previous research. In an RCT (Smits-Engelsman, Jelsma & Ferguson, 2017), 57 children with DCD were prescribed 5 weeks of Wii variable training (including obstacle running and multi-directional mobile games). The children’s BOT-2 sensitivity standard score increased by 0.62. Moreover, the study recorded no statistically notable variation in improvement between the DCD and TD children. These observations suggested that exergames could effectively bridge the sensitivity gap between the DCD and the normally developing populations. In a follow-up RCT by Smits-Engelsman et al. (2018), children with DCD were subjected to 10 weeks of Wii Fit intervention. Resultantly, the children’s 10 × 5 m obstacle running time shortened, and step frequency adjustment speed improved. The report also observed that the enhanced neuromuscular coordination efficiency became the core physiological basis for sensitivity enhancement.

The positive effects of exergames on the sensitivity of children with DCD could be due to two mechanisms. First, some games include scenes, such as sudden stops, directional changes, and multi-directional movements (such as Wii skiing slalom and obstacle running). These scenes can promote the ability to predict movements and improve the efficiency of neuromuscular coordination, which indirectly enhances the muscle activation flexibility and movement switching (Mohammadi, Hadian & Olyaei, 2023). The fun and real-time feedback provided in these games can also encourage training compliance and offer support for gradually forming sensitivity-related skills. Nevertheless, the association requires further research to verify (LeBaron et al., 2022).

Among the limitations of this study were only focusing on DCD and TD children. Therefore, the accumulated data could not be extended to other demographics. Sensitivity results also indicated that the agility index outcomes were unstable following model switching. Consequently, the results related to agility necessitate cautious interpretation. Non-unified research intervention methods presented another restriction to this review, which limited the horizontal comparison of research data. In addition, due to the small sample size, further subgroup analysis of high heterogeneity results cannot be carried out, and this part of high heterogeneity results should also be carefully interpreted. Finally, the mechanism by which video-based exergames improve balance and agility remains unclear, requiring future studies to include more populations, expand evaluation indicators, and improve the evaluation system. Original research could also offer insights into possible mechanisms of such games.

## Conclusion

Video-based exergames present potential benefits for improving the balance of children with DCD. Consequently, such interventions could be a potential non-pharmacological treatment for children with DCD. Nonetheless, the influence of the games on agility, AC, and MD results is still unclear.

### The audience

This study explores the intervention effects of video-based exergames on balance and agility in children with DCD and TD children, and its conclusions hold important academic and practical value for multiple subjects in the field of child motor development and rehabilitation. Pediatric neurologists, rehabilitation therapists and occupational therapists can take it as an evidence-based non-pharmacological intervention scheme to optimize the motor intervention system for children with DCD. Preschool and primary school physical education and special education teachers can reference the findings to integrate video-based exergames into daily teaching. Researchers in child motor development can regard it as a basis for in-depth mechanistic research, and parents of children with DCD can use it as a scientific reference for home motor training.

## Supplemental Information

10.7717/peerj.21664/supp-1 Supplemental Information 1 PRISMA checklist.

10.7717/peerj.21664/supp-2 Supplemental Information 2 Search strategy.

10.7717/peerj.21664/supp-3 Supplemental Information 3 Grade evidence profile.

10.7717/peerj.21664/supp-4 Supplemental Information 4 Sensitivity analysis.

10.7717/peerj.21664/supp-5 Supplemental Information 5 Raw data.

10.7717/peerj.21664/supp-6 Supplemental Information 6 Kappa.

## Additional Information and Declarations

### Data Availability

The following information was supplied regarding data availability:

This is a Systematic review/Meta-analysis.
