# Supplementary material for: The effects of video-based exergames on balance and agility in children with and without developmental coordination disorder: a meta-analysis
Source: PeerJ. 2026 Sep 2;14:e21664. doi: 10.7717/peerj.21664 (PMC13546625; doi:10.7717/peerj.21664)
Supplement: Supplementary file 2 [file peerj-14-21664-s002.docx]

**Supplementary File 1 Search strategy**

| Databases | Search strategy | Result  (Approximately) |
| --- | --- | --- |
| Scopus | #1: Title-Abs-Key (game* OR exergam* OR virtual reality OR virtual therapy OR augmented reality OR virtual environment OR video OR Xbox Kinect OR nintendo OR Wii OR PlayStation)  #2: Title-Abs-Key (developmental coordination disorder OR motor skills disorders)  #3: Title-Abs-Key (child* OR adolescent*)  #4: #1 and #2 and #3  Limiters - Published Date: 20060101-20260130 | 387,861  1,640  2,191,761  82 |
| Pubmed | #1: [Title/Abstract] game* OR exergam* OR virtual reality OR virtual therapy OR augmented reality OR virtual environment OR video OR Xbox Kinect OR nintendo OR Wii OR PlayStation  #2: [Title/Abstract] developmental coordination disorder OR motor skills disorders  #3: [Title/Abstract] child* OR adolescent*  #4: #1 and #2 and #3  Filters: Publication date from 2006/01/01 to 2026/01/30 | 223,378    1,750  1,368,913  112 |
| Web of Science | #1: TOPIC: (game* OR exergam* OR virtual reality OR virtual therapy OR augmented reality OR virtual environment OR video OR Xbox Kinect OR nintendo OR Wii OR PlayStation)  #2: TOPIC: (developmental coordination disorder OR motor skills disorders)  #3: TOPIC: (child* OR adolescent*)  #4: #1 and #2 and #3  Refined by: PUBLICATION YEARS: (20260130-20060101)  Indexes=SCI-EXPANDED, SSCI, CCR-EXPANDED, | 125,509  1,810  571,912  57 |
| EBSCO | #1: Abstract: (game* OR exergam* OR virtual reality OR virtual therapy OR augmented reality OR virtual environment OR video OR Xbox Kinect OR nintendo OR Wii OR PlayStation)  #2: Abstract: (developmental coordination disorder OR motor skills disorders)  #3: Abstract: (child* OR adolescent*)  #4: #1 and #2 and #3  Year: 20060101-20260130 | 215,617  1,461  1,076,442  51 |
