# Supplementary material for: The effects of video-based exergames on balance and agility in children with and without developmental coordination disorder: a meta-analysis
Source: PeerJ. 2026 Sep 2;14:e21664. doi: 10.7717/peerj.21664 (PMC13546625; doi:10.7717/peerj.21664)
Supplement: Supplementary file 3 [file peerj-14-21664-s003.docx]

**Supplementary File 2 Grade evidence profile**

| **Certainty assessment** | | | | | | | **№ of patients** | | **Effect** | | **Certainty** | **Importance** |
| --- | --- | --- | --- | --- | --- | --- | --- | --- | --- | --- | --- | --- |
| **№ of studies** | **Study design** | **Risk of bias** | **Inconsistency** | **Indirectness** | **Imprecision** | **Other considerations** | **Theory-based interventions** | **Routine care** | **Relative (95% CI)** | **Absolute (95% CI)** |  |  |
| Balance | | | | | | | | | | | | |
| 13 | randomised trials | not serious | not serious | not serious | serious^c^ | none | 277 | 269 | - | SMD **0.83 higher** (0.65 higher to 1.01 higher) | ⨁⨁⨁◯ Moderate |  |
| Agility | | | | | | | | | | | | |
| 6 | randomised trials | not serious | serious^b^ | not serious | serious^c^ | none | 133 | 125 | - | SMD **0.69 higher** (0.21 higher to 1.17 higher) | ⨁⨁◯◯ Low |  |
| Aiming and catching | | | | | | | | | | | | |
| 4 | randomised trials | not serious | serious^b^ | not serious | serious^c^ | none | 35 | 34 | - | SMD **0.46 higher** (0.39 lower to 1.13 higher) | ⨁⨁◯◯  Low |  |
| Manual Dexterity | | | | | | | | | | | | |
| 3 | randomised trials | serious^a^ | not serious | not serious | serious^c^ | none | 35 | 34 | - | SMD **0.11 higher** (0.36 lower to 0.59 higher) | ⨁⨁⨁◯ Moderate |  |

**CI:** confidence interval; **SMD:** standardised mean difference

a. Downgrade one level for most of the study have unclear or high risk of bias

b. Downgrade one level for the inconsistency due to considerable heterogeneity (I^2^ > 50%)

c. Downgrade one level for the imprecision due to the total sample size does not meet the rules of thumb (at least 400 participants)
