# Supplementary material for: The effects of video-based exergames on balance and agility in children with and without developmental coordination disorder: a meta-analysis
Source: PeerJ. 2026 Sep 2;14:e21664. doi: 10.7717/peerj.21664 (PMC13546625; doi:10.7717/peerj.21664)
Supplement: Supplementary file 4 [file peerj-14-21664-s004.docx]

**Supplementary File 3 Sensitivity analysis**

**Figure S1.** The balance parameter results after switching the random effects model.

**Figure S2.** The balance parameter results after excluding the maximum effect size study.

**Figure S3.** The agility parameter results after switching the fixed effects model.

**Figure S4.** The agility parameter results after excluding the maximum effect size study.
